# Implementation of exon arrays: alternative splicing during T-cell proliferation as determined by whole genome analysis

**DOI:** 10.1186/1471-2164-11-496

**Published:** 2010-09-14

**Authors:** Toni Whistler, Cheng-Feng Chiang, William Lonergan, Mark Hollier, Elizabeth R Unger

**Affiliations:** 1 Chronic Viral Diseases Branch, National Center for Emerging and Zoonotic Infectious Diseases (proposed), Centers for Disease Control and Prevention, Atlanta GA, 30333, USA

## Abstract

**Background:**

The contribution of alternative splicing and isoform expression to cellular response is emerging as an area of considerable interest, and the newly developed exon arrays allow for systematic study of these processes. We use this pilot study to report on the feasibility of exon array implementation looking to replace the 3' *in vitro *transcription expression arrays in our laboratory.

One of the most widely studied models of cellular response is T-cell activation from exogenous stimulation. Microarray studies have contributed to our understanding of key pathways activated during T-cell stimulation. We use this system to examine whole genome transcription and alternate exon usage events that are regulated during lymphocyte proliferation in an attempt to evaluate the exon arrays.

**Results:**

Peripheral blood mononuclear cells form healthy donors were activated using phytohemagglutinin, IL2 and ionomycin and harvested at 5 points over a 7 day period. Flow cytometry measured cell cycle events and the Affymetrix exon array platform was used to identify the gene expression and alternate exon usage changes. Gene expression changes were noted in a total of 2105 transcripts, and alternate exon usage identified in 472 transcript clusters. There was an overlap of 263 transcripts which showed both differential expression and alternate exon usage over time. Gene ontology enrichment analysis showed a broader range of biological changes in biological processes for the differentially expressed genes, which include cell cycle, cell division, cell proliferation, chromosome segregation, cell death, component organization and biogenesis and metabolic process ontologies. The alternate exon usage ontological enrichments are in metabolism and component organization and biogenesis. We focus on alternate exon usage changes in the transcripts of the spliceosome complex. The real-time PCR validation rates were 86% for transcript expression and 71% for alternate exon usage.

**Conclusions:**

This study illustrates that the Exon array technology has the potential to provide information on both transcript expression and isoform usage, with very little increase in expense.

## Background

Transcriptional analysis using microarray technology has provided key insights into the biologic processes involved in lymphocyte proliferation and activation [1-10]. While changes in alternative splicing has also recently been recognized to critically influence these processes [11,12], it has been technically quite challenging to conduct these studies. A new generation of arrays with sufficient feature density to target every known and predicted exon in the human genome has been developed. These exon arrays have the potential to allow the examination of transcript changes combined with alternate exon usage in response to specific stimuli.

In addition to changes in design of the Exon arrays compared to the previous generation 3' *in vitro *transcription (IVT) arrays [13], significant differences in protocols for their use present challenges to laboratories interested in switching platforms. These include RNA labeling methodologies [14], detection calling [15], quality control checks for labeling [16] and CEL file assessment [17], and analytical methodologies [18,19]. We designed a small study to assess exon array implementation and develop best laboratory practices and expertise to facilitate adoption of this technology. This study examines T-cell responsiveness to stimulation with pharmacologic mimics of antigen-receptor signaling with the intent to examine mRNA alternative splicing and transcript expression during proliferation. This model system was selected because methods for activating or differentiating lymphocytes are well understood and the cells can be readily cultured. Most importantly perhaps, comprehensive gene expression profiles have been published allowing cross platform comparisons [20,21].

## Methods

### Subjects

Blood samples were obtained from 4 healthy Caucasian female volunteers, age 46-56 years. Allergies and hay fever were excluded by self-report. Informed consent was obtained from all subjects, and human experimentation guidelines of the US Department of Health and Human Services were followed in the conduct of this research.

### Primary cell isolation and stimulation

Whole blood was collected from each donor in 20 cell preparation tubes (sodium citrate, Becton Dickinson, CA) and the peripheral blood mononuclear cells (PBMCs) purified according to the manufacturer's instructions. Lymphocytes for each donor were pooled, counted, assessed for viability with trypan blue (>98% in all cases), frozen and stored in liquid nitrogen.

Just prior to use the PBMCs from each donor were fast thawed at 37°C and seeded into 5 T25 flasks (one for each time point) at 1.425 × 10^7 ^cells in 14.25 mL Roswell Parks Memorial Institute 1640 (RPMI) medium containing 20% fetal bovine serum. A T25 flask was harvested and assessed at day 0 (prior to stimulation, D0). Cells in all other flasks were stimulated by the addition of phytohemagglutinin (PHA, 1 μg/mL), Interleukin-2 (IL2, 10 U/mL) and ionomycin (1 μM). Flasks, one from each donor, were harvested and assayed on D1, D3, D5 and D7. Cell counts were performed using trypan blue and samples were split for the individual assays. Flow cytometric analyses were done at the time of harvesting, and the 4 × 10^6 ^cells were pelleted, resuspended in 1 ml TRIzol reagent (Invitrogen, CA) and frozen for microarray analysis.

### Flow cytometric analysis

Immunophenotyping was performed at each time point on 5 × 10^5 ^cells to determine the T-cell (CD3-PerCP-Cy5.5), B-cell (CD19-phycoerythrin) and NK cell (CD56-AlexaFluor488, CD16-FITC) subsets. The assay also included activation markers CD69-allophycocyanin (APC) and CD25-APC. Briefly, samples were incubated with appropriate concentrations of monoclonal antibodies (Becton Dickinson, San Jose, CA) for 30 minutes at room temperature in the dark. Cells were then fixed in 4% paraformaldehyde for 10 minutes at 37°C and stored at 4°C until flow cytometric analysis. A second tube of 5 × 10^5 ^cells was stained for the TUNEL assay using the APO-BrdU kit (BD Pharmingen) as recommended by the manufacturer. After assay completion, Hoechst 33342 was added for cell cycle determination and immediately processed on the flow cytometer. Typically 50,000 events were collected. Data was acquired using CellQuest software (version 3.3) (BD Biosciences). Compensation for the assays and all data analysis was performed using FlowJo software (version 7) (TreeStar, Ashland, OR).

### Microarray procedures

Total RNA was extracted from the PBMCs in TRIzol reagent according to the manufacturer's instructions and quality and quantity was assessed using the Agilent 2100 Bioanalyzer. One μg was labeled using the Exon WT Sense Target Labeling Assay (Affymetrix^®^, Santa Clara, CA) including the labeling controls from the GeneChip^® ^Eukaryotic Poly-A RNA Control Kit. Each step of the labeling protocol was monitored using either the Agilent 2100 Bioanalyzer or the Nanodrop spectrophotometer, as specified by Affymetrix [14]. Quality control (QC) criteria included: 1) after the first cycle cRNA cleanup yields >10 μg as measured by Nanodrop; 2) after the second cycle single-strand cDNA cleanup, yields >5.5 μg. Both checkpoints need OD_260/280 _ratios of 1.8 - 2.2; 3) required the fragmented antisense biotin-labeled cDNA to peak at 40-70 bases when run on the Bioanalyzer. Hybridization buffer, Eukaryotic Hybridization Controls (to confirm the sensitivity of hybridization), and OligoB2 controls (used to orient and grid the array) were added to the cDNA fragments just prior to hybridization to the Affymetrix^® ^Human Exon 1.0 ST Array. Hybridization was at 45°C for 17 hours [14]. Following hybridization, the chips were washed and stained with a phycoerythrin-strepavidin conjugate using the GeneChip^® ^Fluidics Station with the FS450-0001 protocol. The chips were scanned using the Affymetrix^® ^GeneChip^® ^Scanner 3000 and the Affymetrix^® ^GeneChip^® ^Operating Software was utilized for the management, sharing and initial processing of the expression data. All data from the 20 exon arrays has been deposited in Array Express under Accession Number E-MEXP-884.

Array quality control was performed using Affymetrix^® ^Expression Console™ (v 1.1) at the transcript level using core-level probe sets. All image plots passed visual inspection. Hybridization controls had all present with signal increases following concentration. Labeling control signal strengths followed the order Lys < Phe < Thr < Dap. Signal histograms and box plots were examined for raw and processed data. These provided a meaningful way to identify arrays with divergent probe intensity distributions relative to other arrays in the study. The 3 summarization metrics relative log expression (RLE), positive vs. negative ROC AUC and MAD-residual mean were all within the parameters suggested by Affymetrix [16,17].

### Data preparation and analysis

Data was pre-processed using Partek Genomic Suite software ((v6.4) Partek Inc., St. Louis, MO) with the core meta-probe set. The configuration consisted of a pre-background adjustment for GC content and probe sequence, Robust Multi-array Analysis [22] for background correction, quantile normalization and probe set summarization using median polishing. All signals were log_2 _transformed. Library files were those specified by Affymetrix (HuEx-1_0-st-v2) and the annotation file version was na29.hg18.

The data analysis workflow for exon arrays is two pronged [18]. For determination of differential expression, probe set information is summarized into gene level information, and the analytical methodologies remain unchanged from those used for 3' IVT array data. Differential expression was calculated using a repeated measure ANOVA with Time as the main effect. The multiple test correction implemented the q-value method [23] to determine a false discovery rate (FDR) < 0.0001.

A second analysis looks at exon level data. In order to obtain meaningful alternate exon usage information, and to decrease the chance of false positives, several filtering steps were included prior to analysis [24]. The presence of absence of exon expression was determined using detection above background (DABG). All probe sets where the DABG p-value < 0.05 were removed from the analysis. All genes represented by fewer than 5 probe sets in the transcript cluster were removed as it is often difficult to interpret alternative exon incorporation patterns with so few markers. Similar reasoning was used to remove genes represented by >40 probe sets. All transcript clusters with high differential exonic expression (> 5-fold change) between groups were removed, as they have a tendency to produce false positive results for alternative splicing [18]. Data were analyzed for alternative splicing by a repeated measure ANOVA with Time × Probe set as the main effect and corresponding interaction. Pair-wise analyses between D0 and the other time points were also performed. It is not clear how best to correct for multiple testing for the alternative-splicing analysis, however, the optimal cutoff value is dependent on the number of targets you wish to see in your final list. The q-value method was used to control the FDR with a cutoff <0.0001 to determine the presence of alternative exon usage. Several investigators have previously suggested that manual review of the gene plots is required to identify forms of alternative splicing and determine the frequency of changes observed in the dataset [18,25,26]. Criteria used for assessment were the same as those described previously [24].

### Biological interpretation

Gene enrichment analysis was used to interpret the biological impact of alternative exon usage and differential expression during lymphocyte proliferation. Gene Ontology (GO) enrichment analysis was undertaken in Partek Genomic Suite using a chi-square test comparing the proportion of the transcript list in an ontology, to the proportion of the background list in that same ontology. Functional groups with >5 genes and an enrichment score > 3 were considered significant.

### Exon array validation by Quantitative real-time polymerase chain reaction (qPCR)

Exon array findings (both expression and alternate exon usage) were validated using qPCR on the seven genes focused on in this study (additional file 1). Validation of alternate exon usage was performed using 2 different primer-probe combinations within the same transcript. One pair to an area that showed isoform variation (D), and a second to an area which showed none (referred to as the "control" (C) primer-probe set). The latter was used as an endogenous normalizer. For transcript expression validation β-Actin was the normalizer gene with the C primer-probe set for the transcript of interest. β-Actin expression did not vary significantly across the samples, allowing it to be used as the normalizer. Primer Express software (version 2.0) was used for the primer-probe design with transcript sequences from the NetAffx website. Probes were 5' labeled with 6-carboxyfluorescein (FAM) and 3' labeled with MGB (minor groove binder) non-fluorescent quencher. Information on primers and probe sets used are in additional file 1. PCR amplification efficiencies (E = 10^-1/slope^) were determined using a 5-step 5 fold dilution standard curve (10 ng to 16 pg) and PBMC total RNA. Initial optimization experiments showed all PCR products were single bands by agarose gel electrophoresis and calibration curves (plotting relative concentrations against the threshold cycle (Ct)) had RSq values (an indicator of line fit) > 0.99 for all primer pairs. Based on the slopes of the standard curves, the amplification efficiencies ranged from 1.8 to 2.0 (additional file 1).

One microgram of RNA was reverse transcribed into cDNA using random hexamers. The 20 μL qPCR reaction contained 1 × ProbeMaster PCR Master Mix (Roche Applied Science, IN), 0.5 μmol/L of each primer, and 5 μL template. The cycling conditions consisted of one cycle at 95°C for 5 min followed by 45 cycles of 95°C × 15 s, and 60°C × 30 s, on the Lightcycler^® ^480 system (Roche Applied Science, IN). All samples, including standards and non-template control were run in triplicate. The Lightcycler^® ^480 software (version 1.50) was used for data analysis. For comparative quantification the ∆∆Ct method was applied [27,28] as it is suitable for a quick estimation of the relative expression ratio. This model presumes optimal and identical amplification efficiencies of target and reference genes.

## Results

### Exon labeling and microarray quality control assessment

The total RNA extracted from PBMCs had RINs > 8.5 and 1 μg was used for rRNA reduction. The percentage reduction ranged from 53-78%, with D0 unstimulated specimens showing an average reduction of 56%, D1 69%, D3 and D5 67%, and D7 61%. The averaged % present calls on the array data were 52, 52 45, 49 and 50 respectively. Quality control check-points outlined in the methodology were easily met. The average yield for the cRNA step ranged from 18.5 to 65.4 μg and the cDNA from 10.4 to18.9 μg. In both cases the lesser quantities were for D1 specimens. All yields were double that required for the next step, and OD_260/280 _ratios were around 2. After fragmentation the peak size was consistently between 70 and 80 bases. The array QC metrics met all those specified in the Affymetrix reference card [16].

### Data filtration

For the expression data all probe sets with a log_2 _signal < 3 were removed, leaving 126,110 probe sets which summarized to 15,395 transcript clusters. For the alternate exon usage analysis, several layers of filtration were used to reduce the number of tests and increase the detection of alternative splicing events [24]. After filtration the data set covered around 3,700 transcripts.

### Lymphocyte stimulation

**Figure 1:**
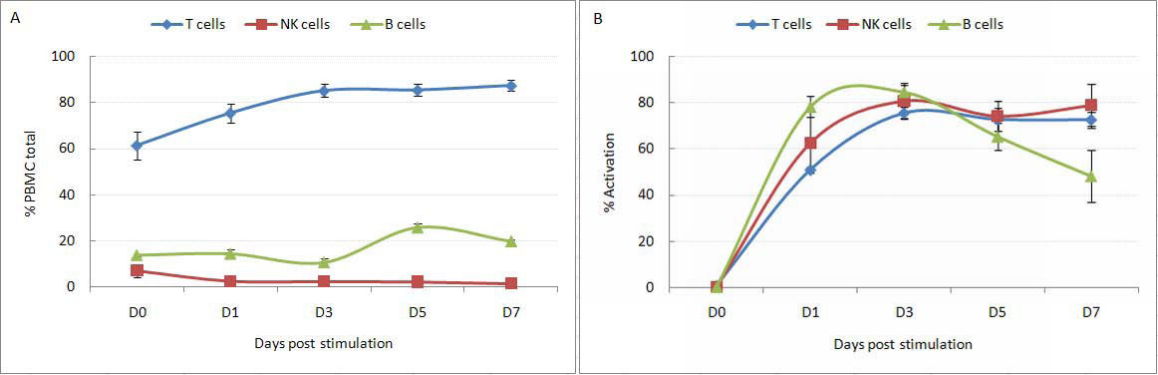
**Results of flow cytometric analysis of PBMC subsets**. T lymphocytes (CD3^+^), B lymphocytes (CD19^+^) and NK cells (CD16^+ ^CD56^+^) showing the averaged percentage of counted events ± standard deviation attributed to each subset (A), and the activation status of each subset determined by CD25 and CD69 status as a % of cell subset numbers ± standard deviation (B).

Stimulation resulted in reduced B- and NK-cell populations, with marked increases in the number of T-cells (Figure 1A). T-cells represent >85% of the cell populations from D3 onward. Up-regulation of cell surface markers CD69 and CD25 reflect the early and late activation of lymphocyte populations respectively (Figure 1B). Activation was first noted by D1 reaching a peak on D3 for NK-, T-, and B-cells, each population showing activation in 70-80% of the population. Total cell numbers decreased from D0 to about 40% for D1 and D3, and then increased on D5 and D7 to 140% of baseline. Stimulated cell viability remained around 90% throughout the time series.

**Figure 2:**
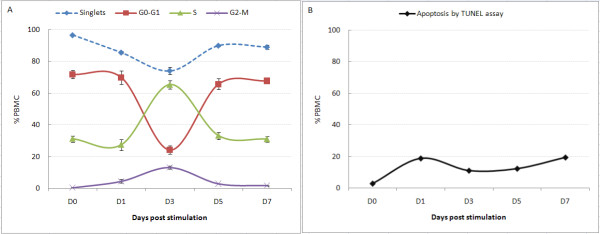
**Cell cycle (A) and apoptotic (B) distribution of lymphocytes after PHA stimulation as determined by flow cytometry**. Cell cycle progression was monitored in PHA-stimulated lymphocytes at indicated times by Hoechst 33342 staining and TUNEL assay for apoptotic status.

Flow cytometric analysis showed the cell cycle position (Figure 2A) and apoptotic status (Figure 2B) of the lymphocyte population across the proliferation assay. A major shift into the S phase was seen by D3, with a small proportion of lymphocytes being at G_2_/M, all returning to G_0_/G_1 _by D5 (Figure 2A). These assays provide good markers for interpreting the differential expression and alternate exon usage data derived from the exon arrays. We are able to overlay biological changes measured by different platforms relating to cell cycle, validating what is being measured by the exon array platform.

### Exon analysis: Alternate exon usage

**Figure 3:**
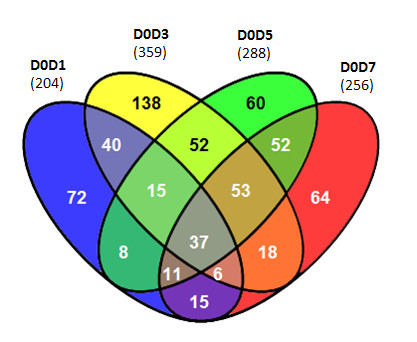
**Venn diagram of transcript clusters with alternate exon usage**. Alternate exon usage identified for transcript clusters by pair-wise ANOVA relative to D0 and confirmed by visual inspection.

Both alternative exon usage and transcript expression profiling were assessed over the 7 days of lymphocyte stimulation. The splice-variant ANOVA exploring alternate exon usage changes over time identified 658 transcript clusters with alternate exon usage after multiple test correction (q-value < 0.0001). Visual inspection [24] recognized 472 transcript clusters as likely candidates. Pair-wise analyses comparing each day to the baseline unstimulated cells (D0) identified 641 unique transcript clusters with alternate exon usage after visual inspection. Venn diagrams show the distribution and overlap of these transcript clusters (Figure 3.)

The 204 transcript clusters showing alternate exon usage within 24 hours of stimulation (D1; Figure 3) were statistically enriched for regulation of signal transduction (18 transcripts). Ten are involved in GTPase mediated signal transduction (see additional file 2), which involves both Ras and Rho subfamilies. Activation of these signaling pathways results in cell growth, differentiation and survival, which are reflected in the D1 enrichment analysis by ontologies for cell differentiation (17 transcripts) and cell death (20 transcripts). Half of the transcripts enriched in the cell death ontology are involved in its negative regulation. Other alternate exon usage changes evident at D1 are two metabolism related categories: Eicosanoid (4 transcripts) and RNA metabolism (20 transcripts, 9 are involved in RNA splicing). D3 alternate exon usage enrichment analysis show RNA metabolism, leukocyte differentiation, and cell death ontologies. However, few transcripts in each category overlap with D1 indicating the dynamics of alternate exon usage are changing. For example, D1 has 9 transcripts associated with RNA splicing and D3, 13. Four are shared between the days (PRPF31, SFRS2, SFRS10 and SFRS10). D3 shows enrichment in signal transduction terms, dominated by T-cell receptor signaling (2 transcripts) and protein kinase cascade (17 transcripts). The later continues through D5. Newly enriched categories by D3 include nuclear transport (9 transcripts, 6 in mRNA export), positive regulation of transcription (9) and DNA repair (13). The latter extends to the D5 (13) and D7 (13) analyses. Three transcripts are common to DNA repair over this time, whereas 6 are unique to D3, 5 to D5 and 5 transcripts to D7 (additional file 2).

Approximately 50% of transcripts showing alternate exon usage across the time series are associated with cellular metabolism.

### Exon analysis: Transcript expression

A parallel analysis identified 2105 unique transcript clusters as differentially expressed (after multiple test correction) during the seven days of lymphocyte stimulation compared to D0. Of these, only 263 (12.5%) also showed alternate exon usage.

### Functional comparison of alternate exon usage and differentially expressed transcript lists

Enrichment analysis of the statistically significant transcript lists compared to those on the array was performed. Examination of "cellular process" in the GO biological process hierarchy (Table 1) allowed us to focus on changes in cell cycle and apoptosis, enabling comparison to the flow cytometric data. Differentially expressed transcripts show a much broader functional role compared to those having alternate exon usage. They are enriched in cell cycle, cell division, cell proliferation, chromosome segregation, cell death, component organization and biogenesis and metabolic process ontologies (Table 1). The enrichment of the alternate exon usage transcript lists only shares the last 2 ontologies.

**Table 1:** Enrichment analysis in GO of transcripts from alternate exon usage and differential expression analysis of lymphocyte proliferation.

Analysis	Compare to D0	No. significant transcript	Cell cycle process	Cell cycle	Cell division	Metabolic process	Chromosome segregation	Cellular organization and biogenesis	Cell proliferation	Cell death	Cell adhesion	Cell communication
No. genes in category			376	381	234	3599	25	924	278	417	620	527

	D1	204										
	D3	359				108				20		
AEU	D5	288	13		9	80		26				
	D7	256	11			75		24				
	Time	472				121		39				
	
	D1	621		30		161			17	25		

	D3	1855	116	119	80	499	12	137	57	66	35	34
DE	D5	880	76	84	61	207	11	68	33	30		
	D7	818	66	68	52	196	10	64	32	32		
	
	Time	1980	120	116	82	518	13	153	53	68		39

Gene Ontology (GO) enrichment analysis of transcript lists was undertaken in Partek Genomic Suite. Results are presented for Cellular Process category under the GO Biological Process. Groups are presented with an enrichment score > 3 and > 5 genes per group.

AEU: alternate exon usage

DE: Differential expression

### Alternate exon usage in transcripts involved in nuclear mRNA splicing via the spliceosome

**Figure 4:**
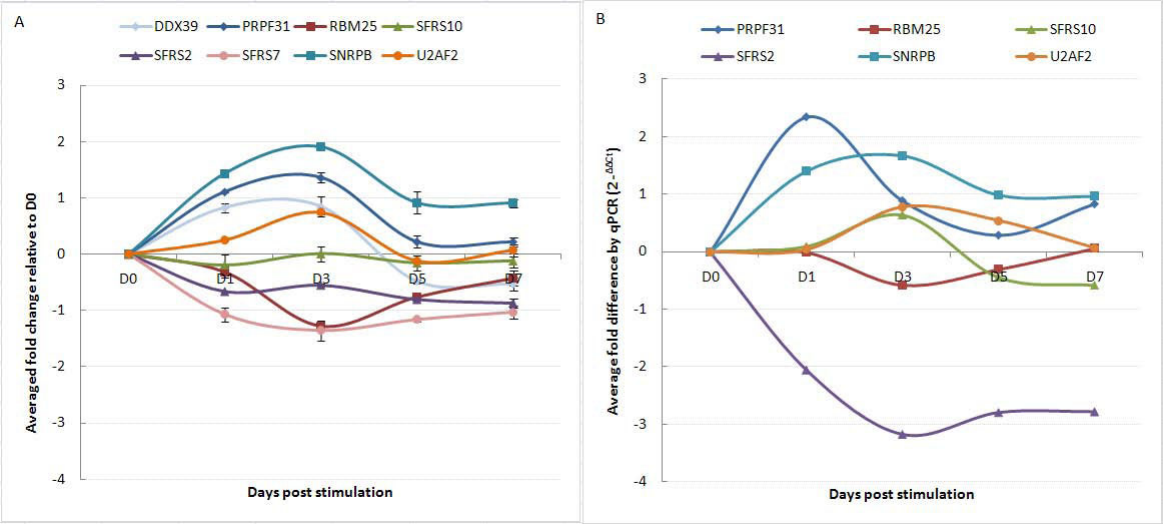
**Expression profiles of transcripts involved in nuclear mRNA splicing via the spliceosome**. (A) Changes in transcript expression levels from exon array data. (B) Validation of array expression data by qPCR. All data is presented relative to D0 baseline.

For the transcripts showing alternate exon usage, RNA processing was a dominant ontology, with nuclear mRNA splicing via the spliceosome being an enriched sub-category. This GO contains 101 transcripts, of which 8 showed alternate exon usage (SFRS2, SFRS7, SFRS10, RBM25, U2AF2, SNRPB, PRPF31 and DDX39). The 3 last transcripts also had statistically significant differential expression (Figure 4A), and a further 15 transcripts showing differential expression only were identified in this ontology.

**Figure 5:**
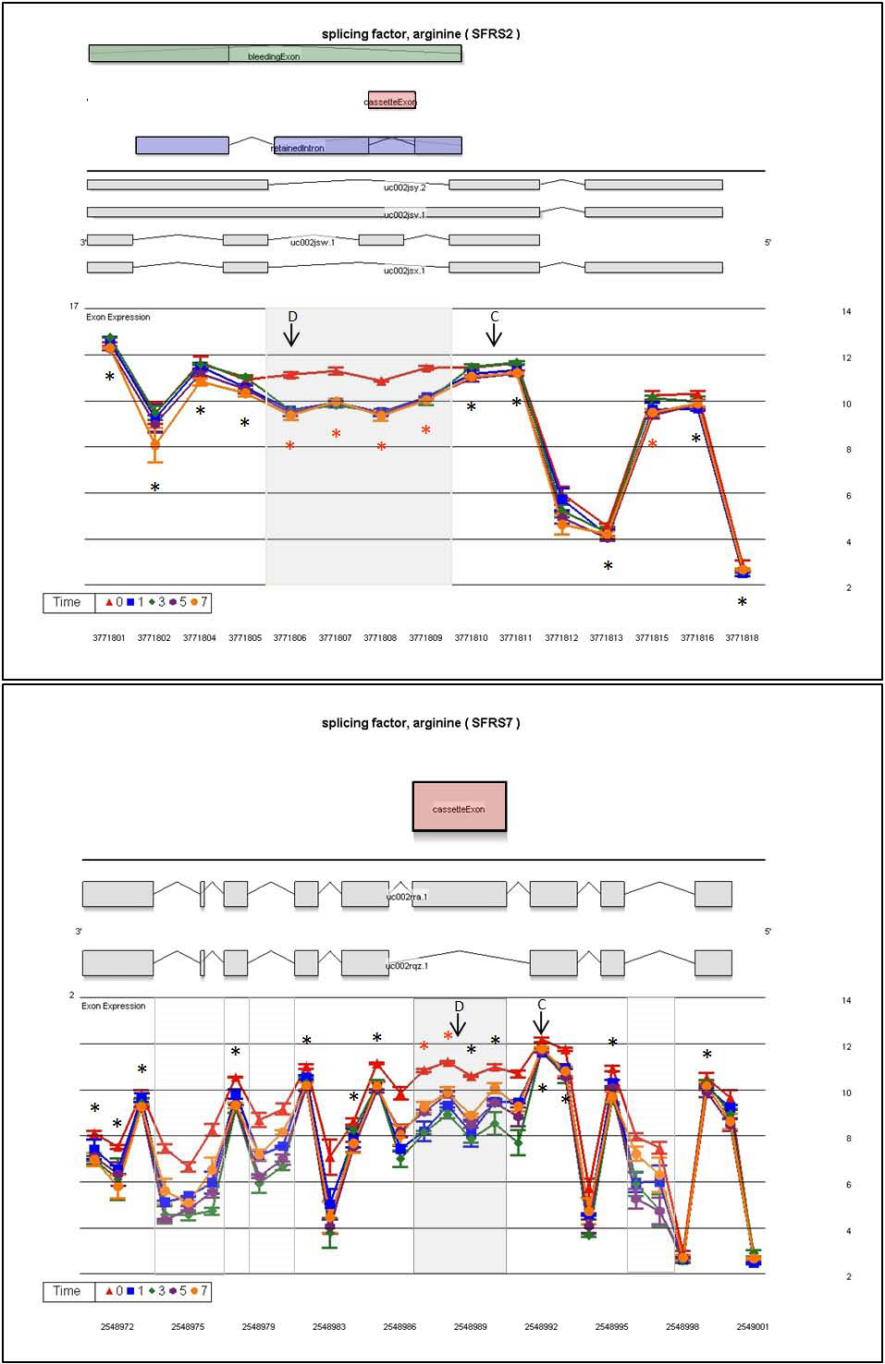
**Transcript cluster gene views showing alternate exon usage by day of proliferation for SFRS2 and SFRS7**. The LS mean log_2 _signal intensity (with standard error bars) for each day is plotted against the full meta-probe set of the transcript. An asterisk (*) denotes probe sets belonging to the core data set, used for analysis, when colored red this indicates statistical significance for alternate exon usage. The top half of the graph shows all known isoforms in this region retrieved from the UCSC browser, the location (above or below the line) defines which DNA strand codes for the transcript. In the alternate location UCSC AltEvent information is given. A green rectangle indicates a bleeding exon; blue a retained intron; and red a cassette exon. Previously described events are highlighted by a grey box and possible new events are denoted by a hatched grey box. Parallel expression lines indicate that the transcript is not differently spliced in the groups. C or D designates the control or differential primer-probe set location for result validation

**Figure 6:**
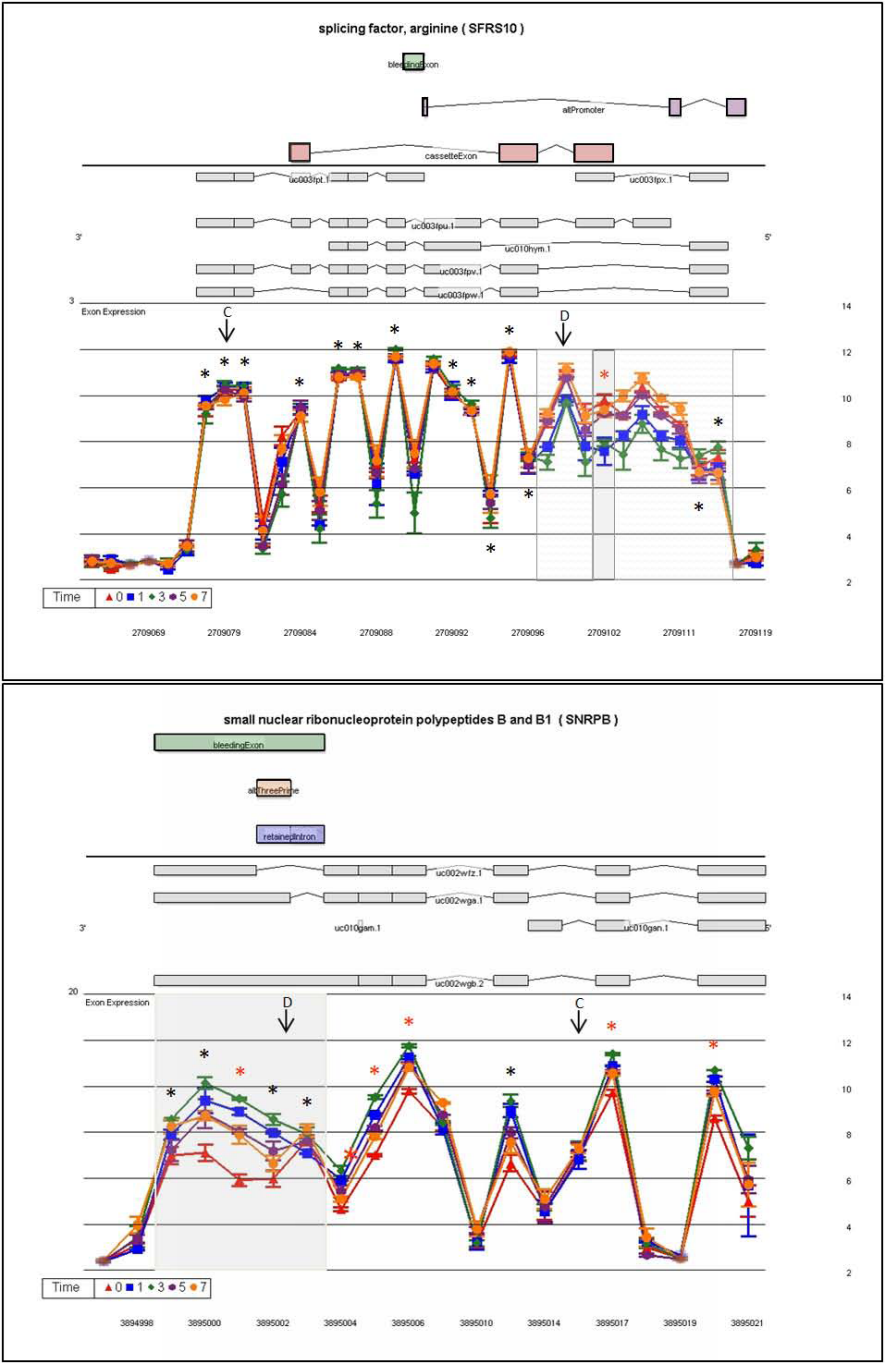
**Transcript cluster gene views showing alternate exon usage by day of proliferation for SFRS10 and SNRPB**. The LS mean log_2 _signal intensity (with standard error bars) for each day is plotted against the full meta-probe set of the transcript. An asterisk (*) denotes probe sets belonging to the core data set, used for analysis, when colored red indicates statistical significance for alternate exon usage. The top section of the graph shows all known isoforms in this region retrieved from the UCSC browser, the location (above or below the line) defines which DNA strand codes for the transcript. In the alternate location UCSC AltEvent information is given. A green rectangle indicates a bleeding exon; blue a retained intron; orange an alternative 3' start site; and red a cassette exon. Previously described events are highlighted by a grey box and possible new events are denoted by a hatched grey box. Parallel expression lines indicate that the transcript is not differently spliced in the groups. C or D designates the control or differential primer-probe set location for result validation.

Several arginine/serine rich splicing factors show different alternative exon usage profiles after lymphocyte stimulation. SFRS2 has four mid-transcript probe sets with significantly higher exon expression on D0 (Figure 5) indicating an increase of the retained intron in unstimulated lymphocytes. SFRS7 shows a mix of mRNA isoforms across the time series (Figure 5) with a cassette exon being highly expressed in unstimulated lymphocytes and decreased inclusion after stimulation. All other core-meta probe sets have similar expression levels on all days. Visualization with the full meta-probe set (to give a clearer picture) indicates 3 areas in SFRS7 with intron retention not described in the UCSC data base (Figure 5). SFRS10 shows statistical significant differences in the exon expression of probe set 2709102 on D1 and D3 relative to D0 (Figure 6). This is confirmed by similar expression patterns for the 7 adjacent probe sets included from the full meta-probe set. Expression levels for the 26 remaining probe sets are the same for all days (Figure 6).

SNRPB (small nuclear ribonucleoprotein polypeptides B and B1) shows major differences in exon expression after stimulation in 5 probe sets at the 3' end of the transcript (Figure 6). This covers an overlapping or "bleeding" exon described in the UCSC AltEvent track descriptor [29].

**Figure 7:**
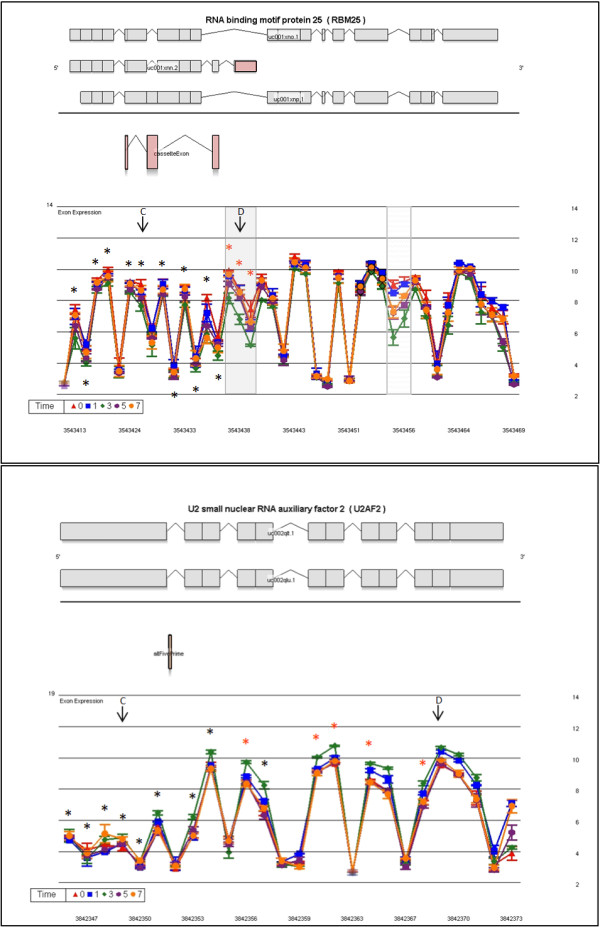
**Transcript cluster gene views showing alternate exon usage by day of proliferation for RBM25 and U2AF2**. The LS mean log_2 _signal intensity (with standard error bars) for each day is plotted against the full meta-probe set of the transcript. An asterisk (*) denotes probe sets belonging to the core data set, used for analysis, when colored red indicates statistical significance for alternate exon usage. The top section of the graph shows all known isoforms in this region retrieved from the UCSC browser, the location (above or below the line) defines which DNA strand codes for the transcript. In the alternate location UCSC AltEvent information is given. An orange rectangle indicates an alternative 3' start site; and red a cassette exon. Previously described events are highlighted by a grey box and possible new events are denoted by a hatched grey box. Parallel expression lines indicate that the transcript is not differently spliced in the groups. C or D designates the control or differential primer-probe set location for result validation

RBM25 (RNA binding motif protein 25) is represented by 15 core meta-probe sets focused on the smallest mRNA isoform (Figure 7). Exon expression levels are statistically significant for 3 probe sets of the 3' cassette exon between D0 and D3. The full meta-probe set adds 28 probe sets belonging to 2 long isoforms. Signal for intronic sequence probe sets (3543455-3543456) indicates intron inclusion, which decreases during cell cycle progression (Figure 7).

U2AF2 (U2 small nuclear RNA auxiliary factor 2) shows different exon expression patterns at each end of the transcript. The 6 probe sets of the 5' exon show no differential expression, but all probe sets thereafter show differential expression (Figure 7). No expression is noted from probe sets to intronic regions.

Gene view diagrams for PRPF31 and DDX39 are given in additional file 3.

### RT-PCR validation of differential transcript expression and transcript alternate exon usage

**Figure 8:**
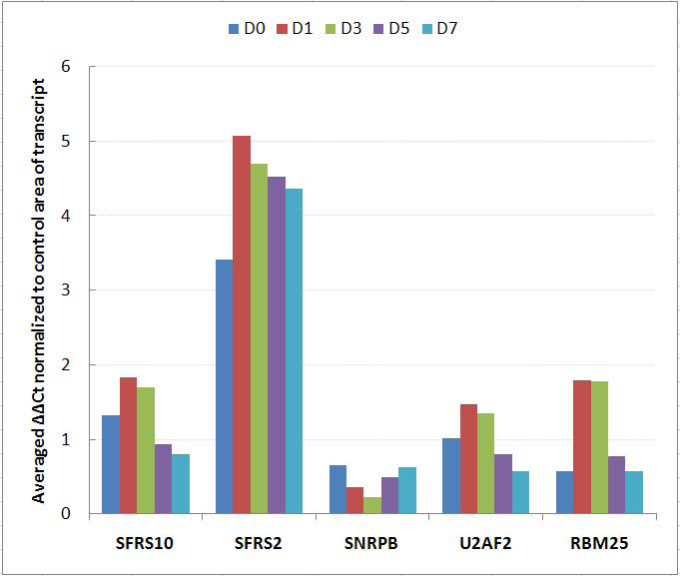
**Validation of alternate exon usage array data by qPCR**. Two primer-probe sets were designed for each transcript, one to an area of constant expression (control, C), the second to region showing alternate exon usage (D). Comparative quantification was calculated using the ∆∆Ct method with the control primer data being used as the endogenous normalizer. Ct levels are inversely proportional to the amount of target RNA in the sample. Comparison of daily profiles with signal intensities in Figure 5-7 shows good result concordance.

Validation was performed on 7 of the 8 transcript clusters discussed above. DDX39 was not included because of difficulties with primer/probe design. Two levels of validation were attempted. The first examined transcript expression across the time series, normalizing expression values to an external gene β-Actin. Six of seven genes were validated, with the microarray results being concordant to the qPCR data in terms of direction of fold changes (Fig. 4A and 4B respectively). Alternate exon usage was validated using an internal transcript area with no differential expression as the normalizer gene. Results reflect the same abundance changes in the area of alternate exon usage (Figure 8) as was evident on the exon arrays (Figure 5, 6, 7). Five of seven events validated (71%). Cross-validation with existing information on alternate exon usage is also extremely useful. We have overlaid the UCSC genome browser AltEvent track onto the relevant gene views (Figure 5, 6, 7). This shows various types of alternative splicing, including alternative promoter usage, cassette exons etc. that result in more than a single transcript. Many of these were concordant with our findings but several events appear to be newly described.

## Discussion

The Affymetrix exon arrays offer a significant increase in content and a greater utility than their 3' expression arrays. The distribution of probes sets across each exon for some 28,800 genes allows the mapping of splice variants. They feature a new design with no mismatch probes, and a random primed protocol to generate sense DNA targets along the entire length of the transcript. These changes raised uncertainty with regard array performance, but transcript expression has been shown comparable with the 3' platforms with close agreement and similar sensitivity [30-32]. The minimal price difference between the platforms makes the exon array an attractive alternative. This pilot study was designed to determine the practicality of implementing these arrays in our laboratory.

Every component of the exon array methodology, from reagents and protocols to quality control and analysis software is different for the exon arrays compared to the previous 3' arrays. Both labeling methodologies take approximately 2 1/2 days with 2 overnight steps (an *in vitro *transcription step followed by hybridization on the second night.) However, the Exon protocol has more steps and requires considerably more hands-on time (we estimate double), especially on the second day. A ribosomal RNA reduction step is required prior to RNA labeling to reduce their impact on amplification and labeling because of the random prime labeling strategy. This necessitates the purchase of special equipment for the magnetic bead separation. A scanner autoloader is also required as scan times increased from 12 to 30 minutes per GeneChip with the change in platform. In our laboratory we were able to do manual labeling of 8 samples per run, whereas for the 3' arrays we could do 24. The downstream processing of data is also a challenge. Data sets are much larger and the analysis is performed at several different levels: exon (for alternate splicing) or transcript (for expression levels) using either the core, full or extended probe sets [13].

The quality control checks for monitoring the labeling protocol and the hybridized GeneChip were extremely useful. The former could save running poor samples on an array, allowing the labeling to be repeated. The latter prevented the inclusion of technical outliers in the statistical analysis. All our samples easily met the QC criteria. The only disparate data were differences in % rRNA reduction. D0 samples had the lowest average reduction (56%), peaking on D1 (69%) and returning to baseline with D3 and D5 at 67%, and D7 at 61%. These differences were most likely due to increased protein synthetic levels expected for cell proliferation.

The corroboration of both transcript expression and alternate exon usage data using a different analytical platform adds weight to the value of exon arrays as an analytical tool. Our validation rates by real-time PCR were 86% for expression data, and 71% for alternate exon usage results. Validation rates for alternate exon usage in other studies using Affymetrix exon arrays range from 21 to 84% [33-35], some of the fluctuation can probably be attributed to differences in data filtration and no visual inspection of the results, emphasizing the importance of best practice methodologies. The alternate exon usage in many of the transcript clusters identified in this study showed greater complexity than a single exon inclusion or exclusion event, illustrating that more than one alternative splice isoform can be maintained concurrently in the mRNA pool. For this data set, it is not possible to dissect if this reflects changes in the ratios of isoforms associated with physiological variation or reflect changes in the cell sub-populations. It is estimated that more than 75% of genes produce alternative transcripts [36,37], contributing to functional diversity in the genome. Therefore there is little question that this is an important component of understanding the complexity of the mammalian transcriptome.

Implementation of the Affymetrix exon arrays requires a considerable input of time but our results show that the benefits are worth the effort. Probably the most challenging area currently, is the downstream analysis of data, mainly because of the increased data set size.

Much work has been done to characterize the genome-wide transcriptional program of lymphocyte activation and proliferation in a wide range of systems, including cell culture [38] whole blood [39], PBMCs [40] and purified cell populations [38,41,42]. This is a critical step toward understanding the biologic processes involved. The interpretation of expression patterns from mixed cell populations is complicated by variation in relative proportions of the cell subsets. We cannot distinguish between genes that undergo modest changes in a large percentage of cells from those that undergo large changes in a small subpopulation of cells. However, the strength of using mixed cell populations is in considering the interactions of these populations. Regulatory functions may be provided by direct cell-to-cell contact or via cytokine secretion from different cells. To understand the regulatory networks underpinning cellular dynamics both purified and mixed-cell populations need to be studied. The same confounders apply to exon array profiling along with the possible differential compartmentalization of nuclear and cytoplasmic isoforms [43].

The majority of transcripts that showed differential expression over time did not show a change in alternate exon usage (87.5%), indicating that distinct networks of regulation are operating. Interestingly, transcripts involving constitutive and alternative splicing regulators (the SR family proteins [40] such as SFRS2, SFRS7 and SFRS10) were significantly enriched for alternate exon usage indicating auto-regulatory organization at the level of transcript splicing. While most of the alternate exon usage events in this study have previously been described, several new splicing patterns were identified, another benefit to implementing the exon array platform.

## Conclusions

The ability to assess the functional state of lymphocytes is important as it relates directly to the ability of people to mount an effective immune response. Differences in transcript abundance are routinely used as indicators of cell activity, and this study shows that both transcript quantity and isoform diversity contribute to the expression complexity of cells. Strategies examining multiple forms of transcript expression changes are likely to provide a broader definition of cell behavior than are studies aimed at singular expression responses. We have demonstrated the utility of the Affymetrix exon arrays and we believe their implementation, in place of expression only platforms, will alter the way we interpret microarray data. As an example, in this study we see different exon usage patterns in several proteins that play a substantial role in regulating alternative splicing by modulating spliceosome assembly and splice site choice [44] after lymphocyte stimulation. This indicates that alternative exon usage plays an important role in lymphocyte proliferation. These results highlight the need for a more rounded view of expression analysis, and show that an extra level of molecular diversity can be added to studies with only a minor increase in cost.

## Competing interests

The authors declare that they have no competing interests.

## Authors' contributions

TW was responsible for the overall design of the study, analysis and manuscript preparation. CFC was responsible for the RNA extraction and generation of exon array data. WL performed the real time qRT-PCR studies and helped in the preparation of the manuscript. MH performed the lymphocyte stimulation experiments and the flow cytometric analysis. ERU contributed to the interpretation of the results and writing of the manuscript. All authors have read and approve the manuscript.

## Supplementary Material

Additional file 1 **Primer probe**. Primer and probe information for qPCR validation of microarray data. Click here for file

Additional file 2 **GO enriched alternate exon usage**. GO enrichment analysis results for transcript lists showing alternate exon usage generated in pair-wise analyses to D0. Click here for file

Additional file 3 **Gene view**. Gene view plots for PRPF31 and DDX39. Click here for file
