# Supplementary figures and images for: Implementation of exon arrays: alternative splicing during T-cell proliferation as determined by whole genome analysis
Source: BMC Genomics. 2010 Sep 14;11:496. doi: 10.1186/1471-2164-11-496 (PMC2996992; doi:10.1186/1471-2164-11-496)

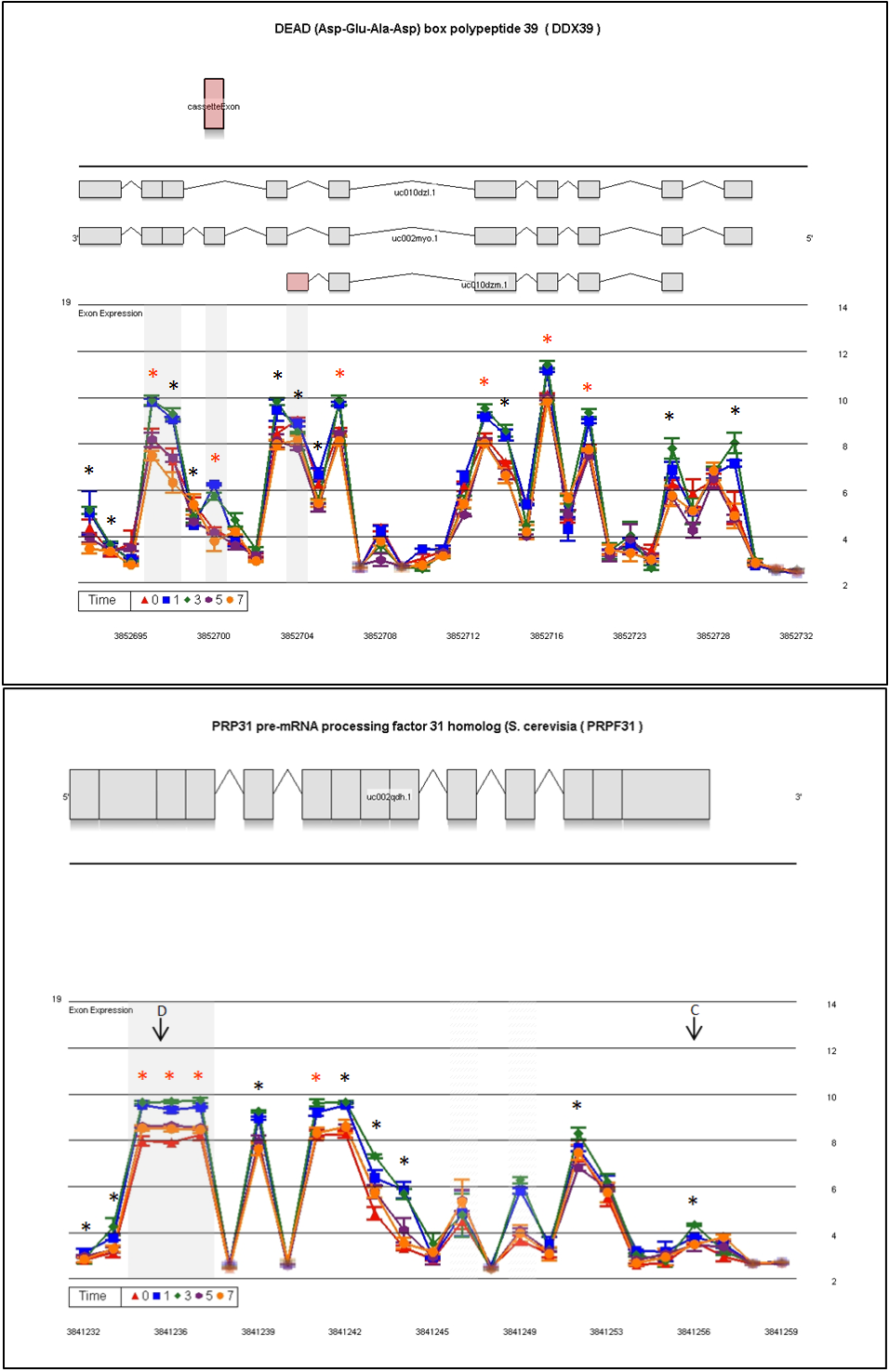

Supplement: Additional file 3 — Gene view. Gene view plots for PRPF31 and DDX39. [file 1471-2164-11-496-S3.TIFF]
